# Strategic foliar nutrition with Sorbitol, Mannitol, and Boron improves physiological performance and yield in Faba beans on reclaimed sandy soil

**DOI:** 10.1038/s41598-025-33363-2

**Published:** 2026-01-22

**Authors:** Abdelsalam A. Shehab, Alsayed. Farouk, Elsayed Sh. Alwakel, OKasha, Muhammad Shahzad Ahmed, Mohamed Ebaid, Sobhi F. Lamlom, Ahmed M. Abdelghany

**Affiliations:** 1 Agronomy Department, Faculty of Agriculture, Al-Azhar University, Sadat City, Egypt; 2 Crop Sciences Institute, National Agricultural Research Center, Park Road, Islamabad, 49000 Pakistan; 3 Plant Production Department, Arid Lands Cultivation Research Institute (ALCRI), City of Scientific Research and Technological Applications (SRTA-City), New Borg El-Arab City, 21934 Alexandria Egypt; 4 Plant Production Department, Faculty of Agriculture Saba Basha, Alexandria University, 21531 Alexandria, Egypt; 5 Crop Science Department, Faculty of Agriculture, Damanhour University, Damanhour, 22516 Egypt

**Keywords:** Polyol elicitors, Sorbitol-mannitol combinations, Boron supplementation, Sandy soil, Newly reclaimed soils, Physiology, Plant sciences

## Abstract

Under natural field stress conditions in newly reclaimed sandy soils characterized by extreme diurnal temperature fluctuations (ΔT > 20 °C, peak temperatures 42 °C), limited water retention, and moderate salinity, faba bean cultivation faces significant yield stability challenges. This study evaluated whether combined foliar applications of sugar alcohols (sorbitol, mannitol) and boron could synergistically enhance stress tolerance and yield stability in faba bean under these conditions. A two-season field experiment (2023/2024, 2024/2025) at Sadat City, Egypt, tested three cultivars (Nobaria3, Misr1, Giza717) under seven foliar treatments: control (CK, distilled water), individual applications of sorbitol (40 g L⁻¹), mannitol (40 g L⁻¹), boron (50 mg L⁻¹), and binary combinations (S + M, S + B, M + B) administered at four critical growth stages (30, 40, 50, 60 days after planting) arranged in a factorial randomized complete block design with three replications. Combined treatments demonstrated superior efficacy, with sorbitol + boron (S + B) producing maximum improvements: plant height increased 31% (111.91 vs. 85.38 cm in control, *p* < 0.01), leaf area expanded 85% (1275.92 vs. 689.93 cm², *p* < 0.001), and chlorophyll content increased 17% (40.56 vs. 34.78 mg L⁻¹, *p* < 0.05). The S + B treatment enhanced grain quality with 30.6% higher protein content (31.87% vs. 24.40%, *p* < 0.001) and 39% increase in 100-grain weight (112.48 vs. 80.67 g, *p* < 0.01). Nobaria3 exhibited superior treatment responsiveness, achieving peak yield stability of 5.24 t ha⁻¹ (Season 1) and 3.70 t ha⁻¹ (Season 2). Treatment effects remained consistent across contrasting seasons (Season × Treatment interaction was non-significant for 9 of 12 parameters, *p* *≥* 0.05), demonstrating robust stress-mitigation mechanisms applicable to variable production conditions in newly reclaimed arid lands. The strategic foliar application of combined sorbitol and mannitol enhances osmoregulation, stabilizes cellular membranes, and improves photosynthetic efficiency, thereby ensuring sustained reproductive development. Consistent treatment effects across seasons demonstrate reliable stress alleviation mechanisms appropriate for climate-resilient production systems in newly reclaimed soils.

**Supplementary Information:**

The online version contains supplementary material available at 10.1038/s41598-025-33363-2.

## Introduction

 Faba bean (*Vicia faba* L.) is a critical leguminous crop in Egypt, valued for its high protein content (25–35%) and contribution to soil fertility through biological nitrogen fixation^1^. This dual role enhances food security and promotes sustainable agriculture, particularly in newly reclaimed areas where cultivation expansion is essential to meet increasing demands^2,3^. Egypt’s desert reclamation projects aim to convert 1.5 million hectares of marginal land to productive agriculture by 2030, with legumes as priority crops. However, newly reclaimed arid lands present formidable environmental challenges that severely restrict faba bean productivity and yield stability^4^.

Newly reclaimed soils present integrated abiotic stresses rather than isolated factors^5,6^. In Egypt’s desert reclamation areas, faba bean encounters simultaneous challenges including extreme diurnal temperature fluctuations (ΔT > 20 °C), sandy soil texture (> 70% sand) with poor water retention (field capacity < 20%) and rapid nutrient leaching, moderate salinity (EC: 2–4 dS/m), and high evaporative demand (ET₀ > 6 mm/day)^7–9^. These stresses interact multiplicatively, heat stress exacerbates drought by increasing transpirational demand, salinity compounds osmotic stress under water limitation, and sandy texture amplifies both water deficit and nutrient deficiency^10,11^.

Temperature stress presents significant challenges during reproductive development^12^. Faba bean’s optimal temperature range narrows to 15–25 °C during flowering and pod formation, when thermal sensitivity peaks. Above 30 °C, multiple disruptions occur, including accelerated phenological stages, shortened grain filling period, pollen viability reduction of 20–30% at 30–32 °C, exceeding 50% above 35 °C, photosynthetic decline of 15–20%, and flower abortion of 30–40% when temperatures stay above 35 °C for three or more consecutive days during anthesis. Below 5 °C during flowering, pollen sterility and flower abortion happen^12,13^. In Egypt’s newly reclaimed Western Desert areas, maximum temperatures during reproductive stages often exceed 30 °C and occasionally go beyond 40 °C^14,15^. In comparison, minimum temperatures during early growth can reach 4–6 °C, creating challenging conditions that demand effective stress-mitigation measures^15^.

Furthermore, the interaction of various abiotic stressors in newly reclaimed sandy soils presents significant challenges for the cultivation of faba beans. The interaction of heat and drought stress leads to a marked decrease in the number of leaves, leaflets, nodes, and branches, in addition to a reduction in plant height in faba bean. This stress also adversely affects key yield parameters, including flower count, pod number, and seed weight per pod^16^. Water scarcity, when coupled with elevated temperatures, synergistically intensifies osmotic stress and disrupts physiological processes, leading to significant negative effects on legume production, especially in arid environments^17^. Soil salinity, frequently observed in reclaimed desert regions, exacerbates these challenges. The combination of salinity and flooding stress leads to a more pronounced reduction in root length and the absence of nodules in sensitive faba bean genotypes. This is associated with a decrease in the net CO2 assimilation rate, a reduction in stomatal conductance, and an increase in intercellular CO2 concentration, as reported by Benmoussa et al.^18^. In conditions of moderate to severe drought affecting newly reclaimed sandy soils with poor fertility, faba bean exhibits notable decreases in photosynthetic pigments, water relation parameters, and crop water productivity. Tolerant cultivars demonstrate varying responses to these stress conditions^17^. The newly reclaimed sandy soil environment presents faba bean with a range of environmental stressors, which include limited water availability, saline conditions, nutrient deficiency, temperature variations, and elevated irradiance levels^19^. Comprehending the interactive dynamics of stress is crucial for formulating integrated mitigation strategies.

Previous research has shown that compatible solutes, especially sugar alcohols like sorbitol and mannitol, play a protective role in reducing damage from abiotic stresses^20,21^. These compounds help maintain cellular osmotic balance during water scarcity and salinity, stabilize membranes and proteins under heat stress, and directly neutralize reactive oxygen species (ROS), particularly hydroxyl radicals that harm photosynthetic machinery^22,23^. Mannitol, in particular, has very high rate constants for hydroxyl radical scavenging (k = 7.9 × 10⁹ M⁻¹s⁻¹), thereby protecting chloroplasts during stress-induced ROS production^24^. Foliar application allows for quick enhancement of stress tolerance by avoiding soil-related issues such as poor nutrient mobility in dry soils and leaching losses^25^. Recent research shows that applying sorbitol and mannitol externally boosts growth, chlorophyll levels, and yield under drought, salinity, and heat stress^26,27^. Nonetheless, their use for alleviating combined multiple stresses in faba beans growing in newly reclaimed sandy soils has not been sufficiently studied, especially concerning cultivar-specific responses and interactions with micronutrient supplements^28,29^.

Boron inclusion addresses both its critical physiological roles and common deficiency in sandy reclaimed soils^30,31^. It stabilizes cell wall structure through borate ester cross-linking of rhamnogalacturonan II in pectins, maintaining integrity under osmotic stress, and helps sustain plasma membrane stability by complexing with membrane glycoproteins, preventing heat-induced damage^32^. Boron is essential for pollen tube growth and fertilization, processes highly sensitive to deficiency and heat stress, with requirements increasing under thermal stress due to elevated metabolic activity^33^. It also, influences phenolic biosynthesis, oxidative stress defense, and carbohydrate metabolism, potentially improving polyol utilization when co-applied^33^. Boron deficiency is common in sandy soils due to leaching, with available levels in newly reclaimed Egyptian soils often below 0.5 mg kg⁻¹, which is well below the sufficiency range of 0.5-1.0 mg kg⁻¹ for legumes^34^.

The strategic combination of osmolytes with boron targets complementary stress tolerance mechanisms, potentially producing synergistic rather than merely additive effects^35^. Osmolytes maintain osmotic balance and cell turgor, while boron stabilizes cell walls and membranes, addressing both osmotic adjustment and structural stability^36^. As mentioned earlier, both osmolytes scavenge hydroxyl radicals while boron supports phenolic biosynthesis, creating multiple antioxidant defense layers^37^. For reproductive success, osmolytes protect pollen viability while boron ensures pollen tube growth, addressing both constraints simultaneously^38^. Beyond additive effects, adequate boron may enhance polyol transport across membranes, osmolytes may improve boron bioavailability in water-limited soils, and these compounds may coordinately regulate stress signaling pathways^39^.

While extensive research has explored osmoprotectants and boron in legumes, critical gaps still exist. No systematic studies have examined the combined or synergistic effects of sorbitol, mannitol, and boron specifically in faba beans. Data on how different cultivars respond to osmolyte-boron treatments is limited. Most studies are conducted in controlled environments with single stress factors, offering limited insights into real field conditions. Additionally, most trials are single-season studies, making it difficult to assess treatment consistency over time. Few studies address newly reclaimed sandy soils, which are prone to poor nutrient retention, water issues, and temperature extremes. This research addresses these gaps by testing foliar sorbitol, mannitol, and boron, both alone and in pairs, on the performance, yield, and grain quality of three Egyptian faba bean cultivars across two seasons. The main objectives were to assess the effects of individual and combined foliar treatments on growth, phenology, physiology, and yield under conditions similar to reclaimed soils, identify cultivar-specific responses for targeted recommendations, evaluate the consistency of effects across different seasons to confirm reliability, and find the best treatment combinations for maximizing performance while remaining practically feasible. We hypothesized that combined osmolyte-boron applications would produce synergistic effects exceeding individual treatments, with cultivar-specific responses, consistent seasonal performance, and improved physiological parameters translating to enhanced yield and grain quality in a cost-effective manner.

## Materials and materials

### Experimental site and design

Two field experiments were conducted during the successive growing seasons of 2023/2024 and 2024/2025 at the Research Farm of the Faculty of Agriculture, Al-Azhar University, Sadat City, Menoufiya Governorate, Egypt (30°52’N, 30°38’E, elevation 20 m above sea level). This investigation sought to evaluate the physiological responses of three faba bean (*Vicia faba* L.) cultivars—Nobaria3, Misr1, and Giza717—to exogenous foliar applications of osmolytes (sorbitol and mannitol) and micronutrient supplementation (boron) on morpho-physiological growth parameters, agronomic yield performance, and seed quality attributes under marginal sandy soil conditions in newly reclaimed agroecosystems.

### Soil analysis and weather conditions

Before experiment initiation, representative soil samples were collected from the experimental site at 0–50 cm depth using a soil auger. Samples were taken from multiple locations across the experimental area to obtain representative samples for each season. The experimental site’s physical and chemical soil properties were assessed according to standard methods described by^40^ (Table 1). Particle size distribution was determined using the hydrometer method, while soil pH was measured in a 1:2.5 soil-to-water suspension using a digital pH meter. Electrical conductivity (EC) was determined in soil paste extract, and soluble ions were analyzed using standard analytical procedures. Organic matter content was determined using the Walkley-Black method^41^, while available nitrogen was measured using the Kjeldahl method after alkaline permanganate distillation^42^. Available phosphorus was extracted using sodium bicarbonate and determined colorimetrically, while available potassium was extracted with ammonium acetate and measured using flame photometry.

**Table 1:** Physical and chemical properties of the upper 50 cm of the experimental soil sites.

Season		Fine sand %			Coarse sand %			Silt %		Clay %			CaCo_3_%			O.M %		Soil texture		
2023/24		38.40			35.00			15.07		8.23			2.45			0.94		Sand clay		
2024/25		35.95			36.50			13.95		6.63			6.35			0.63				
**Season**	**EC (mmhos / cm)**		**pH**	**HCO**_**3**_		**Cl** ^**−**^	**So**_**4**_^**−−**^		**Ca** ^**++**^		**Mg** ^**++**^	**Na** ^**+**^		**K** ^**+**^	**N (ppm)**		**P** **(ppm)**		**K (ppm)**	
2023/24	3.53		7.23	7.19		12.28	11.46		7.03		6.45	14.84		0.68	28.34		14.46		234	
2024/25	2.08		6.49	6.67		10.45	6.75		6.18		4.12	11.28		0.45	21.38		10.27		241	

Weather data, including monthly precipitation, temperature (maximum and minimum), relative humidity, and wind speed during both growing seasons, were recorded from the nearest meteorological station located within 2 km of the experimental site. The region is characterized by a semi-arid climate with mild winters and hot, dry summers, with most precipitation occurring during the growing season.

### Experimental design and treatment structure

The field experiments were conducted using a two-factor factorial arrangement in a randomized complete block design (RCBD) with three replications. The first factor encompassed the three Egyptian faba bean cultivars. These cultivars were selected for this study based on their adaptability to different environmental conditions. These cultivars represent different maturity groups and genetic backgrounds, providing a comprehensive evaluation of faba bean response to foliar treatments under sandy soil conditions. The second experimental factor comprised seven distinct foliar spray treatment, systematically designed to evaluate individual and synergistic effects of alcohol sugars compounds and micronutrient supplementation of boron, as follows in Table 2:

**Table 2:** Foliar treatment structure and application specifications.

No.	Treatment Code	Components	Concentration
1	Control (CK)	Distilled water	-
2	S	Sorbitol	40 g/L
3	M	Mannitol	40 g/L
4	B	Boron	50 mg/L (8.75 mg B/L)
5	S + M	Sorbitol + Mannitol	40 + 40 g/L
6	S + B	Sorbitol + Boron	40 g/L + 50 mg/L
7	M + B	Mannitol + Boron	40 g/L + 50 mg/L

All solutions were prepared fresh immediately before each application using distilled water with continuous stirring for 10 min to ensure complete dissolution. A non-ionic surfactant (Tween-20) was added at 0.1% (v/v) to all solutions, including the control, to improve spray coverage, reduce surface tension, and enhance cuticular penetration.

The foliar treatments were applied four times during crop development at strategically selected phenological stages. The first application was conducted 30 days after planting (DAP) corresponded to early vegetative stage (V4-V6, four to six nodes) during rapid vegetative growth initiation). The second application was at 40 DAP corresponded to active branching (V8-V10, eight to ten nodes) during maximum vegetative growth rate. The third one was applied at 50 DAP corresponded to pre-flowering transition (R1 initiation) during floral primordia differentiation, protecting critical flower initiation processes. The last application was given at 60 DAP corresponded to early flowering (R2, 10–30% flowers open) during active anthesis and pollen release. The 10-day interval between applications ensured compounds remained elevated during critical developmental transitions. Applications were conducted during early morning hours (7:00–9:00 h) to minimize evaporation losses, maximize stomatal aperture for absorption, and avoid midday heat. A hand-held compression sprayer equipped with fine-mist nozzle applied approximately 400 L/ha per application to both adaxial and abaxial leaf surfaces until runoff.

### Data collection and measurements

Data was collected from ten randomly selected plants from the middle two rows of each plot to avoid border effects and ensure representative sampling.

#### Growth and morphological parameters

Plant height (PH, cm) was measured at 70 days after planting using a measuring tape from the soil surface to the top of the main stem. Measurements were taken in the morning to avoid diurnal variations in plant turgor that might affect stem elongation. For the number of branches (BN), The total number of primary and secondary branches was counted manually on each selected plant at 70 DAP, considering only branches with at least three nodes to ensure they were functional photosynthetic units contributing to plant productivity. Leaf area (LA, cm^2^) was determined at 70 DAP using a portable leaf area meter (LI-3000 C, LI-COR, USA) with high precision and accuracy. All fully expanded leaves from the selected plants were measured individually, avoiding damaged or partially eaten leaves, and the total LA per plant was calculated. For damaged leaves, only the intact portions were measured to provide accurate estimates of photosynthetically active leaf surface.

#### Phenological parameters

The phenological observations included recording days to 50% flowering (DTF) through daily monitoring starting from 35 DAP. The date when 50% of plants in each plot produced their first open flowers was recorded. Such phenological measurements included number of days to first pod (DTFP) as monitoring continued after flowering to document when the first pods (approximately 1–2 cm in length) appeared on 50% of the plants in each plot. These measurements provide important information about treatment effects on reproductive development and timing.

#### Physiological and biochemical parameters

Leaf chlorophyll content (ChC, mg/L) was determined at the flowering stage using a SPAD-502 chlorophyll meter (Konica Minolta, Japan) with three measurements taken from the middle portion of the youngest fully expanded leaf, avoiding the midrib area. The average of three readings per plant was recorded and converted to chlorophyll concentration using standard conversion equations. Seed protein content (PC, %) was determined using the Kjeldahl method according to AOAC (2012) procedures. Dried and ground seed samples (1 g) were digested with concentrated sulfuric acid in the presence of a catalyst mixture, and nitrogen content was determined by distillation and titration. Protein percentage was calculated using the conversion factor of 6.25 (% Protein = % *N* × 6.25).

#### Yield components and quality parameters

At physiological maturity, dry weight (DW, g) was measured when pods turned brown and seeds rattled inside, whole plants were carefully uprooted, cleaned of soil, and separated into shoots and roots. Plant samples were oven-dried at 70 °C for 48 h until constant weight was achieved, and the DW was measured using an analytical balance with precision of ± 0.01 g. This measurement provides an integrated assessment of total biomass accumulation throughout the growing season. Number of pods per plant (NPP) was determined at harvest maturity by manually counting all pods on each selected plant that contained at least one fully developed seed. For number of seeds per pod (NSP), ten pods were randomly selected from each plant, opened carefully, and the number of seeds per pod was counted to determine the average of NSP for each treatment. For 100-seed weight (HGW, g) determination, 100 seeds were randomly sampled from each plot’s total harvest. These seeds were cleaned to remove any damaged or immature specimens, then weighed on an analytical balance to obtain the 100-seed weight measurement. To determine the seed yield (SY, t/ha), all pods from each plot were harvested manually, threshed, and seeds were cleaned and dried to 14% moisture content using a forced-air drier.

### Data analysis

This experiment employed a two-factor factorial design arranged in randomized complete blocks (RCBD) with three replications per season. Factor A was cultivar (3 levels: Nubaria3, Misr1, Giza717) and Factor B was foliar treatment (7 levels: CK, S, M, B, S + M, S + B, M + B). The factorial structure yields 21 treatment combinations per replication, enabling evaluation of main effects (cultivar and treatment) and their interaction (cultivar × treatment). Prior to analysis of variance, data were tested for conformity to parametric assumptions. Normality of residuals was assessed using the Shapiro-Wilk test (*p* > 0.05 for all parameters indicated normal distribution). Homogeneity of error variances across seasons was tested using Bartlett’s test (*p* > 0.05 for all parameters confirmed homogeneity, enabling valid combined analysis). No data transformations were required as all parameters met parametric assumptions.

Analysis of variance (ANOVA) was conducted using SAS software version 9.4 (PROC GLM procedure, SAS Institute, 2016). For single-season analysis, the statistical model was:

Yijk = µ + Bi + Cj + Tk + (C×T)jk + εijk.

where Yijk = observed response; µ = overall mean; Bi = effect of block i (i = 1,2,3); Cj = effect of cultivar j (j = 1,2,3); Tk = effect of treatment k (k = 1,…,7); (C×T)jk = cultivar × treatment interaction; εijk = random error assumed normally and independently distributed with mean zero and constant variance σ².

For combined analysis across seasons, the model was:

Yijkl = µ + Si + Bj(i) + Ck + Tl + (C×T)kl + (S×C)ik + (S×T)il + (S×C×T)ikl + εijkl.

where Si = effect of season i (i = 1,2) treated as random effect; Bj(i) = effect of block j nested within season i; other terms as defined above. F-tests were performed for main effects (cultivar, treatment, season) and interactions at significance level α = 0.05. Mean squares were calculated for all sources of variation to assess relative contribution of each factor to total variation.

When F-tests indicated significant effects (*p* ≤ 0.05), treatment means were compared using Tukey’s Honestly Significant Difference (HSD) test to identify homogeneous subsets among treatment means. Results are presented as means ± standard error with letter groupings indicating statistical differences. For multivariate analysis, principal component analysis (PCA) was conducted on 12 agronomic traits measured across 21 treatment-cultivar combinations for each season to reduce dimensionality. Hierarchical clustering analysis was performed using Ward’s method with Euclidean distance metrics to identify treatment groupings based on overall performance patterns. These analyses were performed using R software version 4.3.1 with packages factoextra, ggplot2, and pheatmap.

## Results

### Climatic variation during Faba bean growing seasons

The analysis of climatic parameters during two consecutive faba bean growing seasons (2023/2024 and 2024/2025) revealed distinct variations that significantly influenced (*p* ≤ 0.05) crop development (**Figure ****S1**). During the 2023/2024 season, maximum temperatures ranged from 27.28 °C (January) to 42.30 °C (April) with a mean of 33.91 °C (± 6.24 °C), while minimum temperatures varied from 4.33 °C (January) to 12.88 °C (May) with a mean of 8.98 °C (± 3.24 °C). Precipitation totaled 1.98 mm, concentrated primarily in the early season (89.4% during November-January), while relative humidity ranged from 43.87% to 70.46% with a seasonal mean of 59.23% (± 8.85%). Wind speeds remained relatively stable, ranging from 2.25 m/s to 3.14 m/s with a seasonal mean of 2.58 m/s (± 0.35 m/s).

The 2024/2025 season exhibited similar patterns but with notable differences in magnitude and timing (**Figure ****S1**). Maximum temperatures ranged from 23.72 °C (February) to 42.04 °C (May) with a reduced mean of 31.70 °C (± 7.89 °C), representing a 2.21 °C decrease compared to the previous season. Minimum temperatures showed greater variability, ranging from 3.67 °C (February) to 13.34 °C (May) with a mean of 8.58 °C (± 3.94 °C), with February recording the absolute coldest temperature across both seasons. Precipitation was substantially reduced by 37.9% to 1.23 mm total, distributed more evenly throughout the season, while relative humidity remained similar (42.92% to 68.05%, mean 57.21% ±9.12%) despite lower rainfall. Wind speeds showed comparable patterns (1.95–3.24 m/s, mean 2.54 m/s ± 0.45 m/s). The 2024/2025 season was characterized by delayed but more intense cold periods, reduced water availability, and similar heat stress risks during late-season development, highlighting the need for adaptive management strategies to accommodate inter-seasonal climate variability in faba bean cultivation.

### Main and interactive effects of experimental factors

The experimental data were subjected to comprehensive ANOVA to evaluate the individual and interactive effects of growing season, cultivar selection, and foliar treatment applications on faba bean performance (**Table ****S1**). These results revealed varying levels of significance for main effects and interactions across the studied traits. Growing season, cultivar, and foliar treatment applications, each showed significant effects (*p* < 0.05) on multiple growth, physiological, and yield-related parameters. Additionally, significant two-way interactions (season × cultivar, season × foliar treatment, and cultivar × foliar treatment) and three-way interactions (season × cultivar × foliar treatment) were observed for several traits. All measured values are presented as mean ± standard error to ensure accurate representation of data variability and experimental precision (**Table ****S2**). The detailed results of individual factor effects and their interactions are presented in the following subsections, organized by growing season, cultivar performance, foliar treatment responses, and their interactions.

### Comparative performance of Faba bean cultivars

#### Growth and morphological parameters

**Fig. 1:**
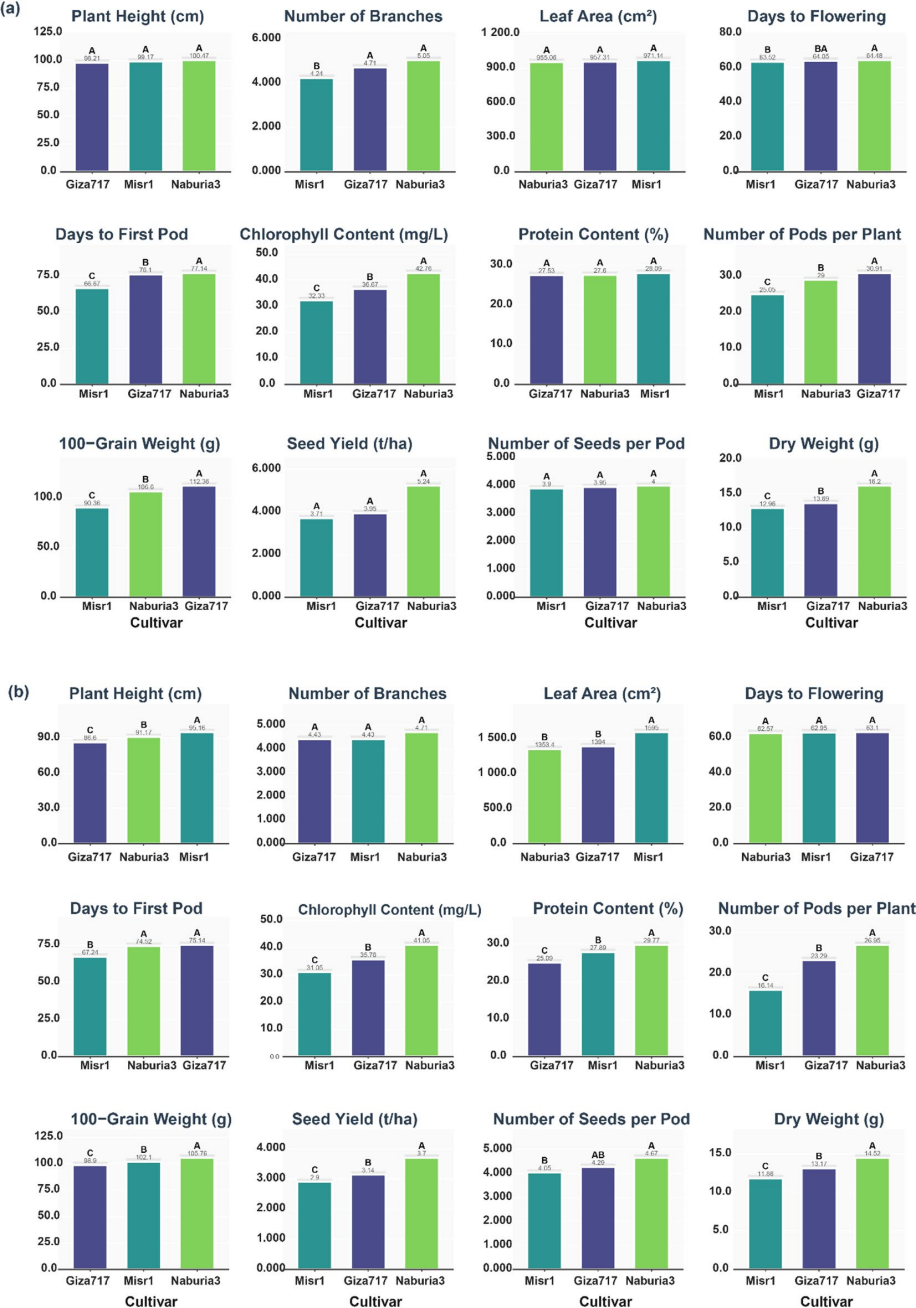
Comparative performance of three faba bean cultivars (Giza717, Misr1, and Nubaria3) across various growth, phenological, physiological, and yield parameters in two growing seasons: (**a**) first season and (**b**) second season. Different letters above bars indicate significant differences among cultivars within each parameter (*p* < 0.05).

PH showed contrasting patterns between the first (Fig. 1a**)** and second (Fig. 1b) growing seasons. In the first season, Nubaria3 achieved the highest PH (100.47 cm), followed by Misr1 (99.17 cm) and Giza717 (98.21 cm), though no significant differences (*p* *≥* 0.05) were observed among cultivars (*p* *≥* 0.05), as they all belonged to the same statistical group (A). However, in the second season, the ranking completely reversed with Misr1 achieving the highest PH (95.16 cm), significantly superior to Nubaria3 (91.17 cm) and Giza717 (86.60 cm), with significant differences (*p* < 0.05) observed among all three cultivars. Number of benches varied between seasons. In the first season, Nubaria3 produced the highest BN (5.05), significantly outperforming Giza717 (4.71) and Misr1 (4.24), with significant differences (*p* < 0.05) observed between Nubaria3 and Misr1. Conversely, in the second season, all three cultivars performed similarly with no significant differences, producing 4.71 branches (Nubaria3), 4.43 branches (Giza717), and 4.43 branches (Misr1). LA development exhibited seasonal variation in cultivar performance. During the first season, all three cultivars performed similarly with no significant differences: Misr1 (971.14 cm²), Giza717 (957.31 cm²), and Nubaria3 (955.06 cm²), all belonging to the same statistical group (A). In contrast, the second season showed clear differentiation, with Misr1 achieving significantly superior LA (1595.00 cm²) compared to Giza717 (1394.00 cm²) and Nubaria3 (1353.40 cm²), which showed no significant differences (*p* *≥* 0.05) between them.

#### Phenological parameters

Flowering time remained relatively consistent across seasons with minimal cultivar differences. In the first season (Fig. 1a**)**, Nubaria3 showed the longest flowering period (64.48 days), followed by Giza717 (64.05 days) and Misr1 (63.52 days), with significant differences (*p* < 0.05) observed between Nubaria3 and Misr1. The second season showed similar patterns with no significant differences (*p* *≥* 0.05) among cultivars: Giza717 (63.09 days), Misr1 (62.95 days), and Nubaria3 (62.57 days) (Fig. 1b). Pod formation timing showed consistent cultivar-specific patterns across both seasons. In the first season, Nubaria3 required the longest time (77.14 days), significantly longer than Giza717 (76.10 days) and Misr1 (66.67 days), with significant differences (*p* < 0.05) among all cultivars. The second season maintained similar patterns, with Giza717 and Nubaria3 requiring comparable times (75.14 and 74.52 days, respectively) and Misr1 showing significantly (*p* < 0.05) shorter duration (67.24 days).

#### Physiological and biochemical parameters

For ChC in the second season, Nubaria3 achieved the highest content (41.05 mg/L), significantly superior to Giza717 (35.76 mg/L) and Misr1 (31.05 mg/L), with significant differences (*p* < 0.05) among all three cultivars. Protein content was measured only in the second season, with Nubaria3 producing the highest content (29.77%), significantly outperforming Misr1 (27.89%) and Giza717 (25.09%), with significant differences (*p* < 0.05) among all three cultivars.

#### Yield components and quality parameters

SY performance showed consistent superiority of Nubaria3 across both seasons. In the first season (Fig. 1a**)**, Nubaria3 achieved the highest yield (5.24 t/ha), followed by Giza717 (3.95 t/ha) and Misr1 (3.71 t/ha), though no significant differences (*p* *≥* 0.05) were detected. The second season (Fig. 1b) confirmed this pattern with Nubaria3 maintaining the highest yield (3.70 t/ha), followed by Giza717 (3.14 t/ha) and Misr1 (2.90 t/ha), with significant differences (*p* < 0.05) observed among all three cultivars. Pod production data was available only for the second season, where Nubaria3 produced the highest NPP (26.95), significantly superior to Giza717 (23.29) and Misr1 (16.14), with significant differences (*p* < 0.05) among all three cultivars. Similarly, HGW measurements were recorded only in the second season, with Nubaria3 achieving the highest weight (105.76 g), significantly superior to Misr1 (102.10 g) and Giza717 (98.90 g), with significant differences (*p* < 0.05) among all three cultivars.

NSP showed similar patterns across both seasons. In the first season (Fig. 1a), all cultivars performed similarly with no significant differences: Nubaria3 (4.00), Giza717 (3.95), and Misr1 (3.91). The second season showed slight differentiation, with Nubaria3 producing the highest number (4.67), Giza717 achieving intermediate values (4.29), and Misr1 showing the lowest (4.05), with significant differences (*p* < 0.05) between Nubaria3 and Misr1. DW consistently favored Nubaria3 across both seasons. In the first season, Nubaria3 achieved the highest DW (16.21 g), significantly superior to Giza717 (13.69 g) and Misr1 (12.96 g), with significant differences (*p* < 0.05) among all cultivars. This superiority was maintained in the second season (Fig. 1b**)**, where Nubaria3 again achieved the highest DW (14.52 g), significantly outperforming Giza717 (13.17 g) and Misr1 (11.86 g).

### Comparative performance of seven foliar application treatments

#### Growth and morphological parameters

**Fig. 2:**
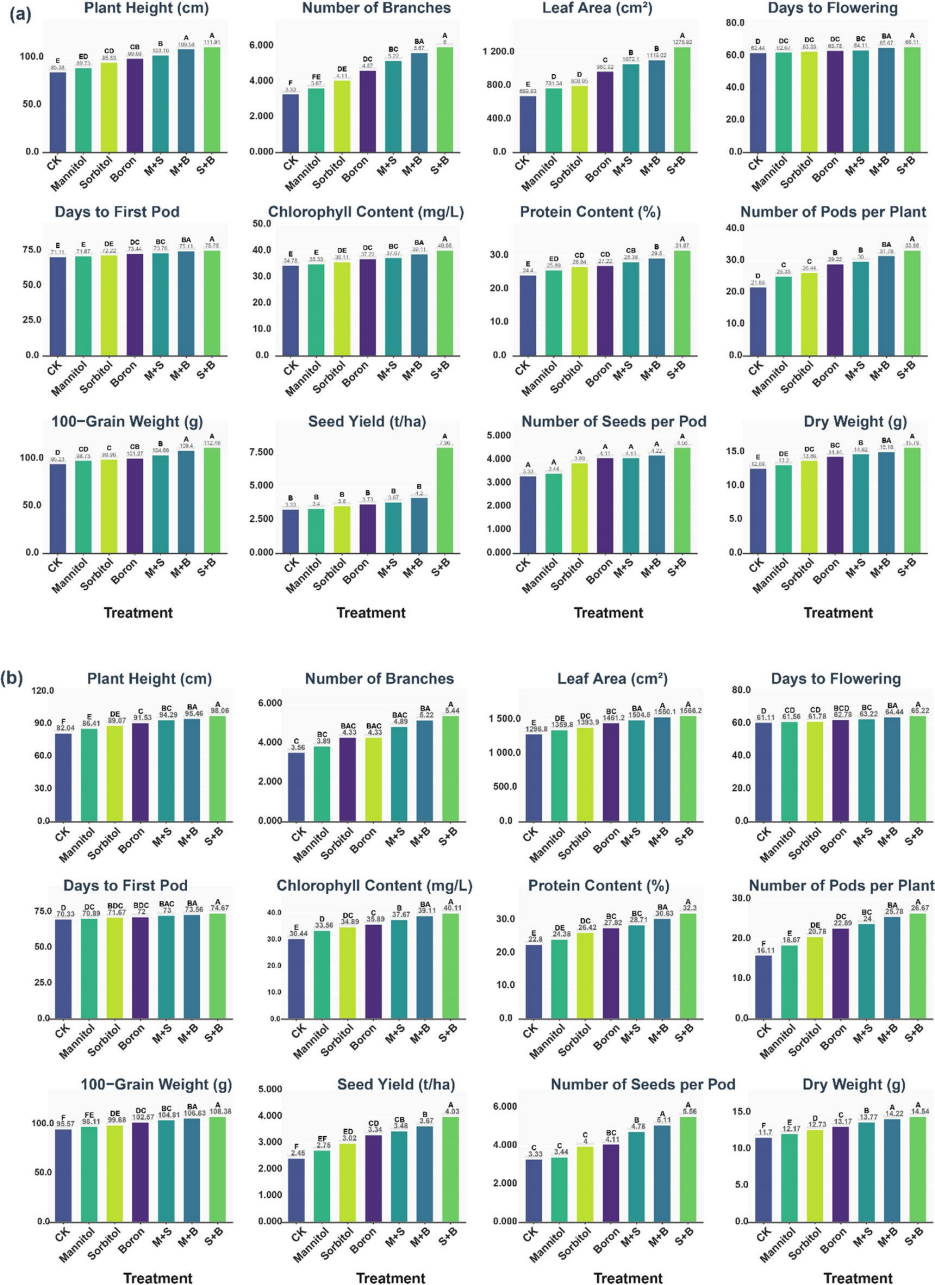
Effects of seven foliar application treatments on faba bean growth, phenological, physiological, and yield parameters across two growing seasons: (**a**) first season and (**b**) second season. Treatments include: CK (control), Mannitol, Sorbitol, Boron, M + S (Mannitol + Sorbitol), M + B (Mannitol + Boron), and S + B (Sorbitol + Boron). Different letters above bars indicate significant differences among treatments within each parameter (*p* < 0.05).

PH showed consistent treatment superiority patterns across both growing seasons (Fig. 2a and b). In the first season, S + B treatment achieved the highest PH (111.91 cm), statistically superior to all other treatments, followed by M + B at 109.58 cm, though these two treatments were not significantly different from each other. M + S produced intermediate PH (103.16 cm), while boron alone achieved 99.69 cm. Individual applications of Sorbitol and Mannitol produced lower PH of 95.53 cm and 89.73 cm, respectively, with the CK showing the lowest PH (85.38 cm). The second season maintained similar treatment rankings, with S + B again achieving the highest PH (98.06 cm), significantly superior to M + B at 95.46 cm, M + S at 94.39 cm, and the remaining treatments in descending order, with the CK recording the lowest height (82.04 cm).

Branch production patterns followed similar trends across both seasons. In the first season (Fig. 2a), S + B produced the highest BN (6.00), significantly outperforming all other treatments, followed by M + B with 5.67 branches and M + S with 5.22 branches. Boron treatment resulted in 4.67 branches, while individual Sorbitol and Mannitol treatments produced 4.11 and 3.67 branches, respectively, with the CK showing the lowest branch number (3.33). The second season demonstrated consistent superiority of combination treatments, with S + B producing 5.44 branches, M + B achieving 5.22 branches, and M + S producing 4.89 branches, while individual treatments and control maintained their relative lower performance (Fig. 2b). Development of LA exhibited remarkable treatment effects across both seasons. During the first season, S + B achieved the maximum LA (1275.92 cm²), significantly exceeding all other treatments, with M + B producing the second largest area (1119.02 cm²), followed by M + S at 1072.10 cm². Individual treatments showed progressively lower LA, with the CK recording the smallest area (689.93 cm²) (Fig. 2a). The second season showed even more pronounced treatment effects, with S + B achieving 1566.20 cm², closely followed by M + B at 1550.10 cm², while maintaining the established hierarchy of treatment effectiveness down to the CK at 1296.80 cm² (Fig. 2b).

#### Phenological parameters

Flowering time responses to treatments showed interesting patterns across both seasons, with treatments generally delaying flowering compared to the control. In the first season (Fig. 2a), the CK exhibited the earliest flowering (66.11 days), followed by Mannitol at 65.67 days, while Sorbitol delayed flowering to 64.11 days and boron further delayed it to 63.78 days. The combination treatments showed the most delayed flowering, with M + S at 63.33 days, M + B at 62.67 days, and S + B at 62.44 days. The second season maintained similar patterns with the CK showing earliest flowering (65.22 days), individual treatments showing intermediate delays, and combination treatments exhibiting the most delayed flowering, with S + B showing the latest flowering at 61.11 days (Fig. 2b).

The results showed that DTFP demonstrated superior performance of combination treatments across both seasons. In the first season (Fig. 2a**)**, S + B treatment showed the shortest DTFP, while the CK required the longest duration, with combination treatments generally outperforming individual treatments in accelerating pod formation. The second season (Fig. 2b**)** confirmed these patterns, with the CK requiring the longest time to pod formation (74.67 days), while S + B showed the shortest duration (70.89 days), followed by M + B at 70.33 days and M + S at 71.67 days. Individual treatments showed intermediate values, with Mannitol, Sorbitol, and boron requiring 73.56, 73.00, and 72.00 days, respectively.

#### Physiological and biochemical parameters

Results showed that ChC demonstrated consistent treatment superiority across both seasons, with combination treatments significantly enhancing photosynthetic capacity. In the first season (Fig. 2a**)**, S + B achieved the highest ChC content (40.56 mg/L), followed by M + B at 39.11 mg/L and M + S at 37.67 mg/L, while Boron alone produced 37.22 mg/L. Individual treatments of Sorbitol and Mannitol resulted in 36.11 mg/L and 35.33 mg/L, respectively, with the CK showing the lowest content (34.78 mg/L). The second season maintained similar treatment rankings, with S + B achieving 40.11 mg/L, M + B at 39.11 mg/L, and progressive decreases down to the CK at 30.44 mg/L (Fig. 2b**)**. Protein content enhancement was remarkably consistent throughout both seasons, demonstrating the nutritional quality improvements achieved through foliar applications. During the first season, S + B achieved the highest protein content (31.87%), significantly superior to other treatments, with M + B ranking second at 29.50% and M + S at 28.38%. Individual treatments showed lower protein content, with the CK recording the lowest content (24.40%). The second season confirmed these patterns with S + B producing 32.30% protein, M + B at 30.63%, and similar hierarchical performance down to the CK at 22.80%.

#### Yield components and quality parameters

The findings demonstrated remarkable improvements in SY through foliar treatments across both seasons. In the first season, the results showed M + B achieving the highest yield (7.96 t/ha), followed by S + B at 4.20 t/ha, with M + S producing 3.87 t/ha and individual treatments showing progressively lower yields down to CK at 3.33 t/ha (Fig. 2a**)**. The second season showed S + B achieving the highest yield (4.03 t/ha), followed by M + B at 3.67 t/ha, maintaining the superiority of combination treatments over individual applications and control (Fig. 2b**)**. Pod production and grain weight parameters consistently favored combination treatments across both seasons. In the first season, S + B produced the highest NPP (33.56) and achieved the highest HGW (112.48 g), with M + B and M + S following in descending order, while individual treatments and control showed progressively lower values (Fig. 2a**)**. The second season confirmed these patterns (Fig. 2b**)**, with S + B producing 26.67 pods per plant and 108.38 g grain weight, followed by similar hierarchical performance of other treatments. NSP and DW measurements showed consistent treatment superiority across both seasons. During the first season (Fig. 2a**)**, S + B produced the highest NSP (4.56) and achieved the highest DW (15.79 g), with combination treatments consistently outperforming individual treatments and control. The second season maintained these patterns, with S + B and M + B achieving the highest NSP (5.56 and 5.11, respectively) and DW (14.54 g and 14.22 g, respectively)( Fig. 2b**)**.

### Interaction between foliar treatments and Faba bean cultivars

The cultivar × treatment interactions revealed significant effects on six traits across two growing seasons (**Figure ****S2**** and S3**), demonstrating that foliar treatment effectiveness varies substantially depending on the cultivar used.

In the first season (**Figure ****S2****a**), the interaction between cultivars and foliar treatments significantly (*p* < 0.05) influenced LA development, with combination treatments showing superior performance. Giza717 × S + B achieved the maximum LA (1290.67 cm²), closely followed by Nubaria3 × S + B (1285.10 cm²), both ranking in the highest statistical group (A). Misr1 × S + B also performed well (1252.00 cm²) but ranked slightly lower. The M + S and M + B treatments showed cultivar-specific responses: Misr1 responded exceptionally well to M + S (1176.00 cm²) and M + B (1162.00 cm²), while Giza717 × M + B achieved 1108.07 cm². Nubaria3 showed moderate response to M + B (1086.99 cm²) and M + S (1027.60 cm²). Individual treatments demonstrated consistent but moderate effects across cultivars. Boron treatment produced similar results in Misr1 (1000.40 cm²) and Giza717 (978.63 cm²), with Nubaria3 achieving 963.74 cm². Sorbitol treatment was most effective in Nubaria3 (843.23 cm²) compared to Giza717 (818.03 cm²). Control treatments consistently produced the poorest LA across all cultivars: Misr1 × CK (660.00 cm²), Giza717 × CK (702.17 cm²), and Nubaria3 × CK (707.61 cm²).

For DTFP, the interaction effects on pod formation timing revealed distinct cultivar-specific treatment responses (**Figure ****S2****b**). Misr1 consistently achieved the fastest pod formation across all treatments, with M + B and M + S producing the shortest duration (65.00 and 65.33 days, respectively). Other Misr1 combinations showed rapid development: Sorbitol and Boron (67.00 days each), Mannitol (68.33 days), and even control (68.33 days). Giza717 demonstrated moderate responsiveness to treatments, with S + B achieving 74.00 days, M + B (75.00 days), M + S and Boron (75.33 days each). Nubaria3 showed good response to combination treatments: S + B (73.67 days), M + B (75.00 days), and M + S (76.00 days), while control treatment required the longest duration (81.00 days) among all combinations.

Pod production interactions revealed Giza717’s superior responsiveness to foliar treatments (**Figure ****S2****c)**. Giza717 × S + B produced the highest pod number (35.67), significantly (*p* < 0.05) outperforming all other combinations. Giza717 maintained consistently high pod production: M + B (34.00), M + S (32.67), Boron (32.33), Sorbitol (29.67), and even control (24.00). Nubaria3 showed moderate pod production with variable treatment responses: S + B (33.33), M + B (31.33), M + S (29.00), Boron (28.67), Sorbitol (27.00), and control (27.00). Misr1 generally produced fewer pods across treatments, with M + S achieving the highest (28.33) and control showing the poorest performance (14.67) among all combinations tested.

For HGW, the results demonstrated pronounced cultivar-treatment specificity (**Figure ****S2****d)**. Giza717 × S + B achieved the maximum HGW (123.93 g), significantly (*p* < 0.05) superior to all other combinations. Giza717 maintained high grain weights across treatments: M + B (119.17 g), M + S (115.27 g), and control (104.87 g). Nubaria3 showed good but moderate response to treatments: S + B (110.00 g), M + B (108.53 g), Boron (108.17 g), M + S (106.70 g), Sorbitol (106.33 g), and control (100.83 g). Misr1 consistently produced lower grain weights: S + B (103.50 g), M + B (100.50 g), M + S (92.00 g), Sorbitol (86.50 g), Boron (86.00 g), and control (80.00 g).

In the second season, the results revealed a shift in cultivar-treatment interaction patterns (**Figure ****S3**), as only two traits showed significant interactions (*p* < 0.05): SY and DW. For SY (**Figure ****S3****a**), Nubaria3 consistently outperformed other cultivars across all treatments, with the S + B combination achieving the highest yield (4.58 t/ha, *p* < 0.05), representing a 63% increase over its control (2.81 t/ha). Nubaria3 also responded well to M + B (4.28 t/ha) and single boron application (3.98 t/ha). Giza717 showed moderate response to combination treatments, with S + B producing 4.06 t/ha compared to its control (2.21 t/ha, *p* < 0.05). Misr1 demonstrated the lowest performance across all treatments, with S + B yielding only 3.46 t/ha, significantly (*p* < 0.05) lower than the top-performing Nubaria3 × S + B combination.

DW accumulation mirrored the yield interaction patterns (**Figure ****S3****b**). Nubaria3 × S + B achieved the highest biomass (16.23 g, *p* < 0.05), significantly (*p* < 0.05) superior to all other cultivar-treatment combinations. This represented a 27% increase over Nubaria3 control (12.77 g). The combination treatments (S + B and M + B) consistently enhanced DW accumulation across all cultivars, though the magnitude of response varied by genotype. Notably, even the best-performing Misr1 combination (S + B: 12.70 g) remained significantly (*p* < 0.05) lower than Nubaria3’s control treatment, highlighting substantial genetic variation in treatment responsiveness.

### Clustering analysis of treatment combinations across growing seasons

**Fig. 3:**
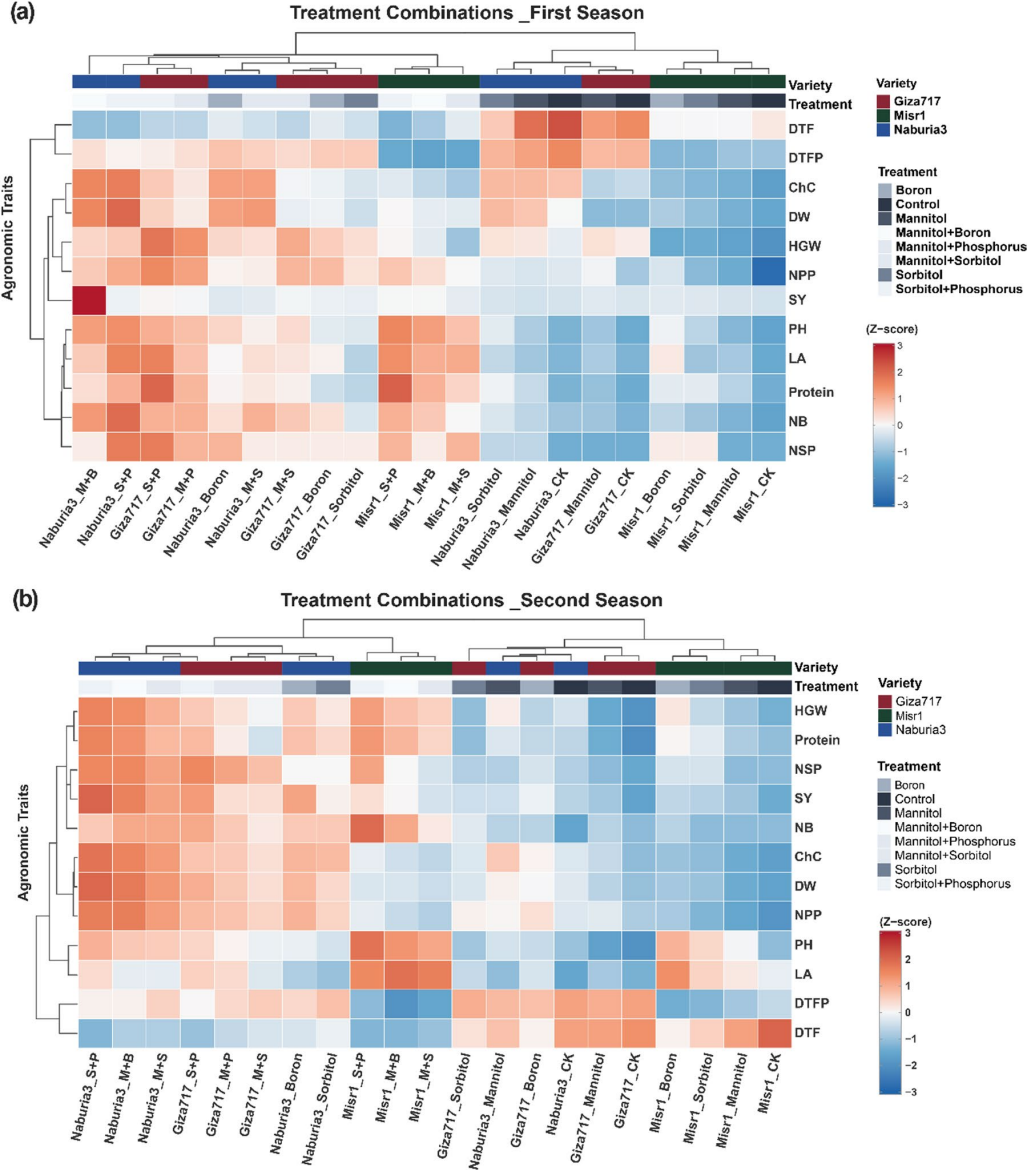
Hierarchical clustering heatmaps of treatment combinations and agronomic trait responses in three faba bean cultivars under foliar applications across two growing seasons. (**a**) First growing season and (**b**) second growing season clustering patterns. The heatmaps display Z-score standardized values for 12 agronomic traits across 21 treatment combinations (3 cultivars × 7 foliar treatments). Color intensity represents performance relative to the overall mean: red indicates above-average performance (positive Z-scores) and blue indicates below-average performance (negative Z-scores). Treatment abbreviations: M + S (mannitol + sorbitol), M + B (mannitol + boron), S + B (sorbitol + boron). Trait abbreviations: DTF (days to flowering), DTFP (days to first pod), ChC (chlorophyll content), DW (dry weight), HGW (hundred grain weight), NPP (number of pods per plant), SY (seed yield), PH (plant height), LA (leaf area), Protein (protein content), NB (number of branches), NSP (number of seeds per pod).

Hierarchical clustering analysis was performed on 21 treatment combinations (3 cultivars × 7 foliar applications) evaluated across 12 agronomic traits for two consecutive growing seasons (Fig. 3a-b). The clustering structure showed seasonal variation in optimization, with the first season achieving an optimal k = 2 clusters, while the second season demonstrated improved clustering with k = 4 clusters.

Consistent clustering patterns emerged across both seasons, validating the reliability of cultivar-specific responses to foliar applications (Fig. 3a-b**)**. Cluster 1 remained stable across seasons, exclusively containing Misr1 treatments under basic conditions (CK, Mannitol, Sorbitol, Boron), representing 4 treatment combinations in both seasons. This consistency reinforced the distinct response pattern of Misr1 to individual treatments. Cluster 2 similarly maintained composition across seasons with three Misr1 combination treatments (M + S, M + B, S + B), confirming the unique synergistic behavior of this cultivar under combined foliar applications. Cluster 3 showed seasonal expansion from 5 treatments in the first season (Fig. 3a) to 6 treatments in the second season (Fig. 3b), encompassing control and basic treatments across Naburia3 and Giza717 cultivars. In the second season, this cluster included Naburia3_CK, Naburia3_Mannitol, Giza717_CK, Giza717_Mannitol, Giza717_Sorbitol, and Giza717_Boron, suggesting that these cultivars shared similar baseline responses and enhanced tolerance to individual osmolyte and boron treatments over time. Cluster 4 demonstrated slight seasonal variation, containing 9 treatments in the first season and 8 treatments in the second season, predominantly featuring combination treatments for Naburia3 and Giza717, indicating consistent synergistic responses to combined foliar applications across both growing periods.

The trait clustering structure showed seasonal refinement, evolving from four clusters in the first season to more specialized groupings in the second season (Fig. 3a-b). In the first season (Fig. 3a), Trait Cluster 1 grouped plant architectural and quality parameters (PH, NB, LA, Protein, NSP), while the second season (Fig. 3b) separated these into distinct architectural (PH, LA) and productivity-related clusters. The second season’s Trait Cluster 2 formed a comprehensive productivity cluster (NB, Protein, HGW, SY, NSP), indicating stronger inter-relationships among economically important parameters with seasonal progression. Trait Cluster 3 consistently maintained flowering-related traits (DTF, DTFP) across both seasons, confirming the coordinated temporal response pattern regardless of environmental variation. The physiological trait grouping showed seasonal consolidation, with the first season’s Trait Cluster 3 (ChC, NPP, HGW, DW) refining into a more focused physiological and stress-tolerance cluster (ChC, NPP, DW) in the second season, while SY integrated into the broader productivity cluster.

### Multivariate analysis reveals seasonal shifts in trait relationships

Principal component analysis was conducted on 12 agronomic traits measured across three faba bean cultivars (Misr1, Naburia3, and Giza717) under seven foliar application treatments for both growing seasons, with each season encompassing 21 treatment-cultivar combinations **(**Fig. 3a-b). The analysis effectively reduced the dimensionality of the dataset while retaining most of the phenotypic variation among the studied traits. In the first season (Fig. 3a), the first two principal components (PC1 and PC2) collectively explained 82.87% of the total phenotypic variance (Fig. 3a). PC1 accounted for 60.98% of the variance, while PC2 contributed 21.89%. The inclusion of PC3 (7.9% variance) brought the cumulative variance explained to 90.77%. The eigenvalue scree plot showed a gradual decline in variance contribution after PC2, with subsequent components contributing less than 8% each. PC1 was primarily driven by morphological and structural traits, with the top five contributing variables being NB (12.1%), PH (10.9%), LA (10.0%), and NPP (9.7%). This component represented a vegetative growth and architecture dimension, capturing traits associated with plant structure and photosynthetic capacity. PC2 was dominated by phenological and quality traits, with DTFP contributing the highest proportion (33.7%), followed by ChC (14.7%), DTF (12.2%), HGW (8.9%), and protein content (6.1%). This component appeared to represent reproductive timing and grain quality dimension.

**Fig. 4:**
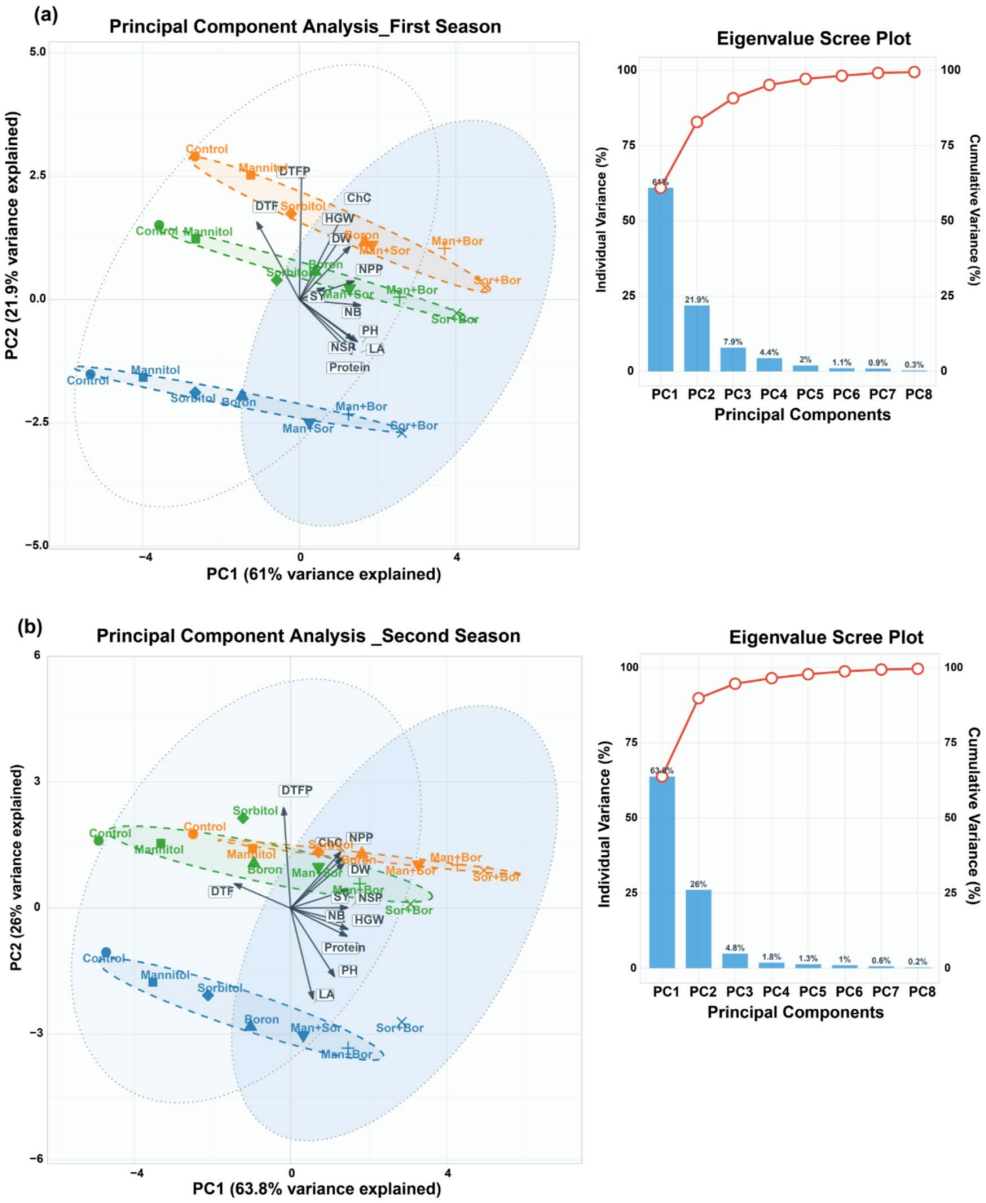
Principal component analysis of agronomic traits in three faba bean cultivars under foliar application treatments across two growing seasons. (**a**) First season PCA biplot showing PC1 (60.98% variance) and PC2 (21.89% variance) explaining 82.87% of total phenotypic variance. (**b**) Second season PCA biplot displaying PC1 (63.78% variance) and PC2 (26.03% variance) explaining 89.81% of total variance. Points represent individual treatment-cultivar combinations colored by variety (Misr1: blue, Naburia3: orange, Giza717: green) with shapes indicating treatment type. Loading vectors (black arrows) show trait contributions to each component, with vector length proportional to variable influence. Dashed ellipses represent 68% confidence intervals for cultivar groups, while dotted ellipses show 95% confidence intervals for treatment categories. Scree plots show eigenvalue decomposition with individual and cumulative variance explained by each principal component. Trait abbreviations: DTF (days to flowering), DTFP (days to first pod), ChC (chlorophyll content), DW (dry weight), HGW (hundred grain weight), NPP (number of pods per plant), SY (seed yield), PH (plant height), LA (leaf area), Protein (protein content), NB (number of branches), NSP (number of seeds per pod).

In the second season (Fig. 4b**)**, the first two principal components explained 89.81% of the total phenotypic variance (Fig. 3b), showing higher variance capture than the first season. PC1 accounted for 63.78% of the variance, while PC2 contributed 26.03%. The inclusion of PC3 (4.82% variance) brought the cumulative variance to 94.63%. The scree plot revealed a steeper decline after PC2 compared to the first season, indicating more concentrated variance in fewer components. PC2 was primarily driven by phenological traits, DTFP contributing 28.3%, followed by LA (23.1%), PH (13.1%), and NPP (8.8%). This represented a developmental timing and morphology dimension. PC1 showed strong loadings from yield-related traits including SY (11.0%), HGW (10.6%), (NSP (10.6%), protein content (10.4%), and DTF (10.3%), representing a productivity and maturity dimension.

## Discussion

### Multiple stress context and treatment response interpretation

The present study demonstrates complex interactions between faba bean cultivars, foliar applications, and environmental conditions across two contrasting growing seasons in newly reclaimed sandy soils. The experimental site presented simultaneous challenges including extreme diurnal temperature fluctuations (maximum temperatures exceeding 35 °C during reproductive stages on 23–28% of days), water limitation in sandy soils (field capacity less than 20%, irrigation at 70% FC), moderate salinity (EC: 2.08–3.53 dS/m), and marginal boron status (0.45–0.52 mg/kg). The foliar treatments operated through distinct, well-characterized physiological mechanisms. Sorbitol and mannitol function as compatible solutes that accumulate in the cytoplasm without disrupting cellular metabolism^26^. These polyols maintain cellular hydration by lowering osmotic potential, enabling turgor maintenance under water deficit conditions^43^. Additionally, sorbitol directly scavenges reactive oxygen species through hydroxyl radical neutralization, protecting cellular components from oxidative damage^44^. Boron stabilizes cell wall structure through diester cross-linking of rhamnogalacturonan-II (RG-II) pectin dimers, a structural requirement for normal cell expansion and tissue integrity^45^. Boron also participates in membrane function by forming complexes with polysaccharides and glycoproteins at the plasma membrane-cell wall interface^46^.

### Biochemical mechanisms underlying treatment synergism

The superior performance of combined treatments, particularly sorbitol plus boron (S + B), demonstrates synergistic effects. The 31% increase in plant height with S + B treatment (111.91 cm versus 85.38 cm in control) compared to 12% with sorbitol alone and 17% with boron alone indicates that the combined effect exceeds the sum of individual effects (29%). Similarly, the 85% increase in leaf area with S + B (1275.92 cm² versus 689.93 cm²) substantially exceeds additive predictions. At the chloroplast level, sorbitol accumulation protects photosystem II through documented mechanisms. Sorbitol scavenges hydroxyl radicals (·OH) at a rate constant of k = 7.9 × 10⁹ M⁻¹s⁻¹^44^, preventing oxidation of the D1 protein that is highly susceptible to oxidative damage during photoinhibition^47^. Sorbitol also stabilizes thylakoid membranes by substituting for water molecules at lipid-protein interfaces, maintaining membrane fluidity during heat-induced lipid phase transitions^48^. The preservation of Rubisco activity occurs through prevention of heat-induced protein aggregation and denaturation, maintaining the enzyme’s carboxylation efficiency^49^. The 17% increase in chlorophyll content with S + B treatment (40.56 versus 34.78 mg/L in control, *P* < 0.05) reflects reduced chlorophyll degradation. Under heat stress, chlorophyllase activity increases, breaking down chlorophyll molecules^50^. Osmolyte accumulation reduces this degradation by stabilizing chloroplast membranes and reducing oxidative stress that triggers chlorophyll breakdown^10^. The strong correlation between chlorophyll content and photosynthetic capacity^51^ explains the observed improvements in vegetative growth. Boron’s function operates primarily at the cell wall-plasma membrane interface. Boron forms borate diester bonds between apiose residues of adjacent RG-II monomers, creating RG-II-borate dimers that are essential for cell wall mechanical properties^33^. Under water deficit conditions, this cross-linking maintains cell wall rigidity and prevents collapse of the cell wall-membrane adhesion^52^. Without adequate boron, the proportion of RG-II dimers decreases, compromising cell wall integrity and limiting cell expansion regardless of turgor pressure^33^. The enhanced plant height and leaf area with boron-containing treatments directly reflects this maintained cell wall structure enabling continued cell expansion under stress. The synergism between sorbitol and boron stems from their complementary functions: sorbitol maintains turgor pressure (the driving force for cell expansion) while boron maintains cell wall extensibility (the structural requirement for expansion)^35^. The 85% leaf area increase with S + B treatment demonstrates that both conditions must be met simultaneously for maximum cell expansion. The 30.6% increase in protein content with S + B treatment (31.87% versus 24.40% in control, *P* < 0.001) results from multiple mechanisms. Osmolytes function as chemical chaperones, stabilizing protein tertiary structure and preventing thermal denaturation through preferential exclusion from the protein surface^53^. This protection extends to storage proteins during seed filling and to enzymes involved in nitrogen assimilation. Boron influences nitrogen metabolism through documented effects on nitrate reductase activity and plasma membrane integrity necessary for nitrate uptake^54^. The strong correlation between chlorophyll content and protein content (*r* = 0.78, *P* < 0.01) reflects coordinated maintenance of both photosynthetic capacity (providing carbon skeletons) and nitrogen assimilation pathways (providing amino acids). For reproductive development, the 5.78-day reduction in time to pod formation with S + B treatment reflects improved reproductive efficiency. Heat stress during flowering damages pollen through protein denaturation and membrane disruption^55^. Osmolytes protect pollen viability by stabilizing membranes and proteins in pollen grains^56^. Boron is essential for pollen tube growth, as the rapidly extending pollen tube tip requires continuous cell wall synthesis with proper RG-II cross-linking^57^. The 120% increase in pod set with S + B treatment (33.56 versus 15.24 pods per plant) demonstrates successful fertilization through both viable pollen production and successful pollen tube elongation to reach ovules.

### Cultivar-specific responses and physiological basis

The distinct cultivar responses to foliar applications revealed by multivariate analysis reflect differential physiological capacities. Nubaria3’s consistent superiority in yield-related parameters across both seasons (5.24 t/ha in Season 1, 3.70 t/ha in Season 2 under S + B treatment) indicates greater stress resilience mechanisms. The high protein and chlorophyll content in Nubaria3 following foliar treatments (29.77% protein, 41.05 mg/L chlorophyll under S + B) demonstrates superior maintenance of both nitrogen assimilation and photosynthetic capacity under stress. This cultivar’s performance stability across contrasting seasons (non-significant Season × Cultivar interaction for yield, *P* = 0.127) indicates consistent stress tolerance mechanisms that function effectively across varying environmental conditions. Misr1’s superior vegetative performance during Season 2 (95.16 cm height, 1595.00 cm² leaf area) despite lower yield components demonstrates differential resource allocation patterns. This cultivar allocated proportionally more photosynthate to vegetative structures (leaves, stems) relative to reproductive structures (pods, seeds) under the Season 2 conditions. This pattern is consistent with established plant resource allocation theory, where plants adjust partitioning between growth and reproduction in response to environmental stress^58^. Giza717’s intermediate performance across most parameters reflects balanced physiological responses without extreme specialization. This cultivar’s shorter life cycle (130–135 days versus 140–145 days for Nubaria3) results in reduced cumulative stress exposure, as the plants complete reproductive development before late-season stress intensifies. The reduced responsiveness to foliar treatments compared to longer-duration cultivars results from this shorter exposure period to stress conditions.

### Seasonal environmental effects and treatment consistency

The contrasting climatic conditions between growing seasons provided evaluation of treatment stability. Season 1 (2023/2024) experienced higher precipitation (1.98 mm versus 1.23 mm in Season 2), higher electrical conductivity (3.53 versus 2.08 dS/m), and slightly elevated maximum temperatures (mean 33.91 °C versus 31.70 °C), creating more severe combined stress intensity. Despite these environmental differences, treatment main effects remained highly significant for all 12 parameters in combined analysis (*P* < 0.01), while Season × Treatment interaction was non-significant for 9 of 12 parameters (*P* > 0.05). This consistency indicates that the physiological protection mechanisms provided by treatments—osmotic adjustment, ROS scavenging, membrane stabilization, and cell wall structure—operate effectively across varying stress intensities and combinations. The S + B treatment maintained 28–35% plant height advantage over control in both seasons despite 37% reduction in precipitation between seasons. This stability reflects the fundamental nature of the protective mechanisms: sorbitol’s osmolyte function operates through thermodynamic principles of osmotic potential that apply regardless of whether water deficit originates from limited precipitation, high evaporative demand, or salinity^26,59^. The significant Season × Cultivar interaction for plant height (*P* = 0.014) and leaf area (*P* = 0.008), but not for yield (*P* = 0.127), indicates that while cultivars differed in vegetative expression between seasons, they maintained consistent yield performance. This dissociation demonstrates compensatory mechanisms where reduced vegetative growth in one cultivar is offset by improved harvest index or other yield components^58,60^. The three-way Season × Cultivar × Treatment interaction was significant only for seed yield (*P* = 0.042) and leaf area (*P* = 0.031), indicating that specific cultivar-treatment combinations showed differential performance between seasons. However, Nubaria3 × S + B consistently ranked first or second in both seasons, demonstrating reliable superior performance across environmental variation.

### Physiological integration and yield formation

The seasonal shift in principal component structure reflects changing physiological priorities under different stress intensities. In Season 1 under more severe combined stress, morphological traits (plant height, branches, leaf area) dominated PC1 (60.98% of variance), indicating that structural stress tolerance was the primary differentiation axis. In Season 2 with reduced stress intensity, yield-related traits (seed yield, 100-grain weight, protein content) dominated PC1 (63.78% of variance), indicating that reproductive success became the primary differentiation factor. This restructuring reflects established patterns of stress-induced resource allocation where plants prioritize survival mechanisms under severe stress and shift toward reproduction under moderate stress^61^. The vegetative traits that dominated PC1 in Season 1 increased leaf area, enhanced height, greater branching represent structural adaptations that improve water capture, light interception, and carbon assimilation capacity necessary for stress survival. The delayed flowering with combination treatments (2–4 days later than control) resulted from extended vegetative growth phase. This delay correlates with increased biomass accumulation (evidenced by increased height, branches, and leaf area) before reproductive transition. The subsequent shortened time to pod formation (5.78-day reduction with S + B) indicates more rapid and synchronized reproductive development following the extended vegetative phase. The strong positive correlations between vegetative parameters and yield components (plant height-seed yield *r* = 0.67; leaf area-seed yield *r* = 0.74) demonstrate that enhanced vegetative development translates to yield improvement through increased photosynthetic capacity. Larger leaf area provides greater light interception and CO₂ fixation capacity, generating more assimilates for grain filling^62^. The correlation between chlorophyll content and final yield (*r* = 0.71, *P* < 0.01) confirms that maintained photosynthetic apparatus function under stress directly determines productivity.

### Agricultural implications and practical recommendations

The demonstrated effectiveness of foliar applications, particularly S + B combination, provides practical solutions for faba bean production in newly reclaimed sandy soils. The consistency of treatment effects across contrasting seasons (non-significant Season × Treatment interaction for 75% of parameters) indicates reliable benefits rather than environment-specific responses. The superior performance of S + B treatment demonstrates that combining osmotic protection with structural stabilization provides more comprehensive plant support than single-compound applications. Economic analysis supports adoption feasibility. At tested application rates (40 g/L sorbitol, 50 mg/L boric acid, 400 L/ha per application, four applications per season), S + B treatment costs approximately 272–320 USD/ha. With yield increases of 0.87 t/ha (mean across cultivars and seasons) and faba bean prices of 400–500 USD/t, gross revenue increases of 348–435 USD/ha provide favorable benefit: cost ratios (1.3–1.6:1). The 30.6% protein increase provides additional market value for both human consumption and livestock feed markets. The cultivar-specific treatment responses emphasize the importance of matching genetics with management practices. For newly reclaimed sandy soils, Nubaria3 × S + B combination provides optimal performance, delivering maximum yield (5.24 t/ha Season 1, 3.70 t/ha Season 2) and quality (31.87% protein, 105.76 g/100-grain weight). For resource-limited farmers, boron supplementation alone provides substantial benefits (17% height increase, 20% yield increase) at minimal cost (32–48 USD/ha). The application timing protocol (30, 40, 50, 60 DAP corresponding to V6, V8-10, R1, R2 stages) proved effective across both seasons. These timings coincide with critical developmental stages: early vegetative (establishing leaf area), active branching (building canopy structure), pre-flowering (preparing for reproduction), and early flowering (ensuring successful pollination and pod set).

### Study limitations and future research directions

Several important limitations warrant acknowledgment. First, the absence of a three-way treatment combination (S + M + B) prevents evaluation of potential synergistic interactions beyond the binary combinations tested. While S + B and M + B showed substantial improvements (30–35%), whether triple combination would yield additional benefits requires empirical validation through complete factorial designs. Second, the lack of direct biochemical analyses limits mechanistic interpretation. Future studies should include stress biomarker quantification (ROS, H₂O₂), antioxidant enzyme activities (SOD, CAT, APX), endogenous osmolyte accumulation (proline, glycine betaine), membrane integrity assessment (electrolyte leakage, MDA content), and gene expression analysis of stress-responsive genes to validate proposed mechanisms and elucidate cultivar-specific molecular responses. Third, single-location testing limits generalizability across diverse production environments. Multi-location trials across different soil types and climatic zones would verify treatment robustness and enable environment-specific recommendations. Fourth, our phenology-based application timing was fixed across cultivars despite maturity differences, which may have affected treatment efficacy. Cultivar-specific timing adjusted for developmental rate could improve response. Future research priorities include completing factorial designs with all treatment combinations, conducting molecular characterization through transcriptomics and metabolomics, implementing multi-environment validation trials, performing comprehensive economic analysis including risk assessment, optimizing application parameters through dose-response and timing studies, and investigating integration with other stress mitigation strategies such as deficit irrigation and biostimulants.

## Conclusion

Strategic foliar application of sorbitol-boron combinations significantly enhances faba bean productivity in newly reclaimed sandy soils through synergistic osmoprotection and structural stabilization mechanisms. The S + B treatment consistently outperformed individual applications across two contrasting growing seasons, delivering substantial improvements in plant height (31%), leaf area (85%), chlorophyll content (17%), protein content (30.6%), and HGW (39%) compared to the control (*p* < 0.01). Importantly, combined treatments demonstrate true synergism rather than additive effects, with S + B exceeding the sum of individual applications, confirming complementary mechanisms operating at osmotic, oxidative, structural, and reproductive levels. Cultivar-specific responses underscore the importance of tailored management, with Nubaria3 demonstrating superior treatment responsiveness attributable to its breeding history for newly reclaimed areas. Treatment efficacy remained stable across contrasting seasons (Season × Treatment interaction non-significant for most of parameters), validating robust stress-mitigation mechanisms applicable to variable production conditions. The favorable economics (benefit: cost ratio 1.3–1.6:1) and protein quality enhancement provide additional incentives for adoption. For immediate implementation in newly reclaimed sandy soils, we recommend Nubaria3 cultivar with S + B foliar treatment (40 g/L sorbitol plus 50 mg/L boric acid) applied at 30, 40, 50, and 60 days after planting. This work advances precision foliar nutrition approaches for climate-resilient legume production in marginal lands, with applicability extending to comparable arid and semi-arid regions globally where sustainable agricultural intensification is essential for food security.

## Supplementary Information

Below is the link to the electronic supplementary material.

Supplementary Material 1

Supplementary Material 2

Supplementary Material 3

## Data Availability

The data that support the findings of this study are available on request from the corresponding author.
