# Supplementary material for: Strategic foliar nutrition with Sorbitol, Mannitol, and Boron improves physiological performance and yield in Faba beans on reclaimed sandy soil
Source: Sci Rep. 2026 Jan 22;16:3262. doi: 10.1038/s41598-025-33363-2 (PMC12834954; doi:10.1038/s41598-025-33363-2)
Supplement: Supplementary file 3 — Supplementary Material 3 [file 41598_2025_33363_MOESM3_ESM.docx]

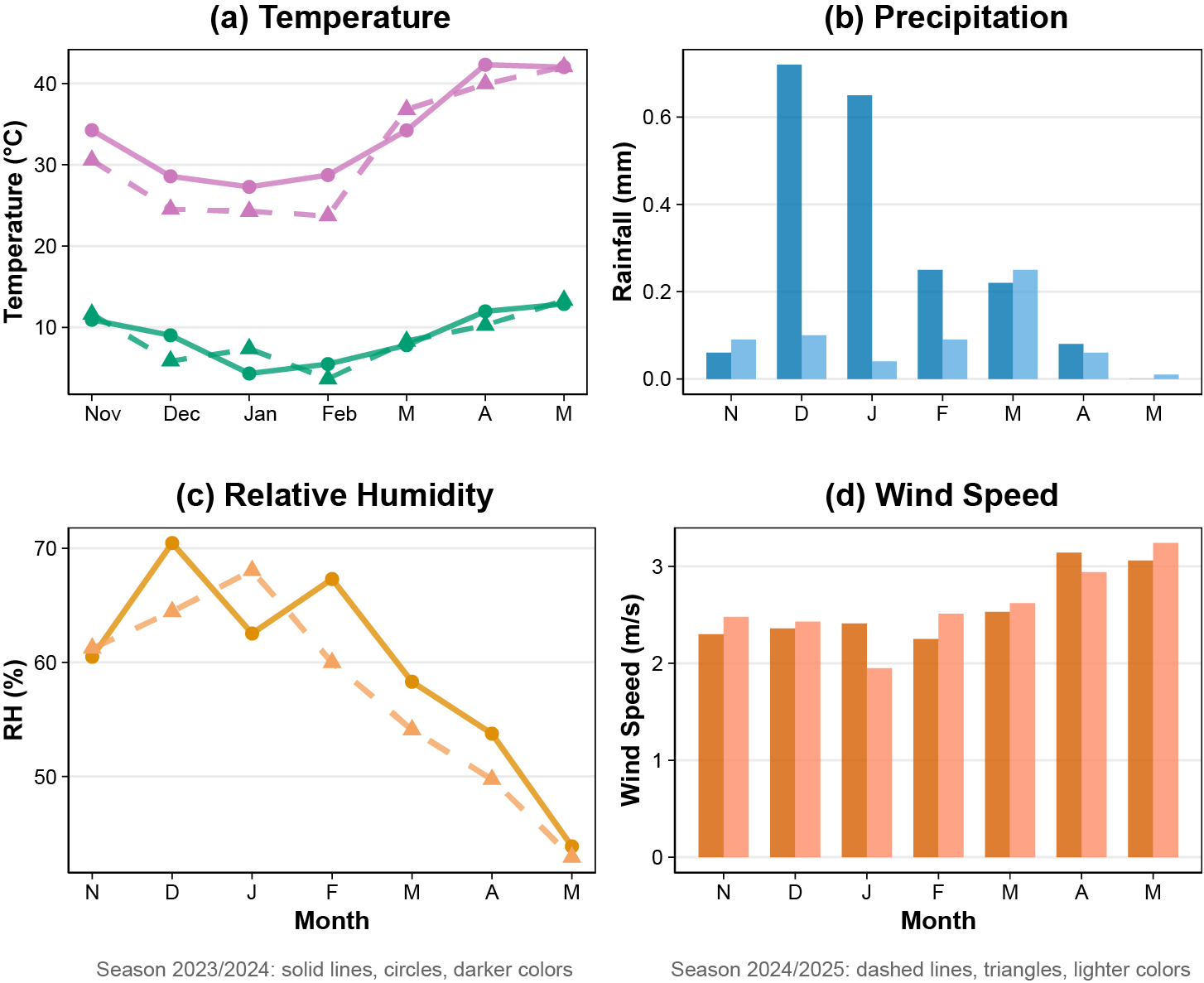


**Figure S1.** Seasonal variation of climatic parameters during two consecutive faba bean growing seasons (2023/2024 and 2024/2025). (a) Temperature patterns showing maximum (upper lines) and minimum (lower lines) temperatures throughout the growing period. (b) Monthly precipitation distribution (mm). (c) Relative humidity (%) (d) Wind speed. Season 2023/2024 is represented by solid lines, circles, and darker colors; Season 2024/2025 is shown with dashed lines, triangles, and lighter colors.


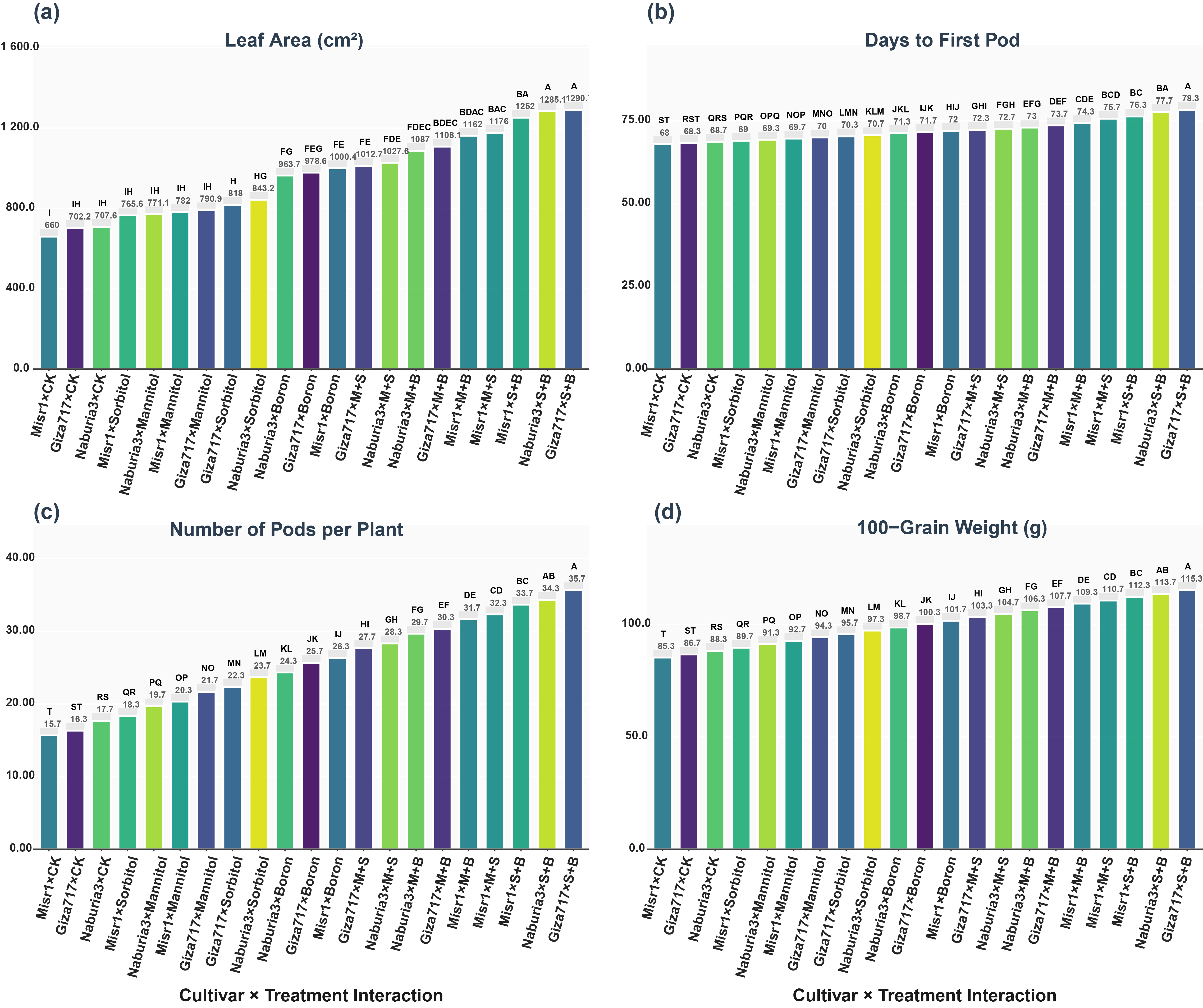


**Figure S2.** Interaction effects between three faba bean cultivars (Misr1, Giza717, Nubaria3) and seven foliar application treatments on selected parameters during the first growing season: (**a**) leaf area (cm²), (**b**) days to first pod formation, (**c**) number of pods per plant, and (**d**) 100-grain weight (g). Treatments include: CK (control), M (Mannitol), S (Sorbitol), B (Boron), M+S (Mannitol + Sorbitol), M+B (Mannitol + Boron), and S+B (Sorbitol + Boron). Different letters above bars indicate significant differences among cultivar × treatment combinations within each parameter (p < 0.05).


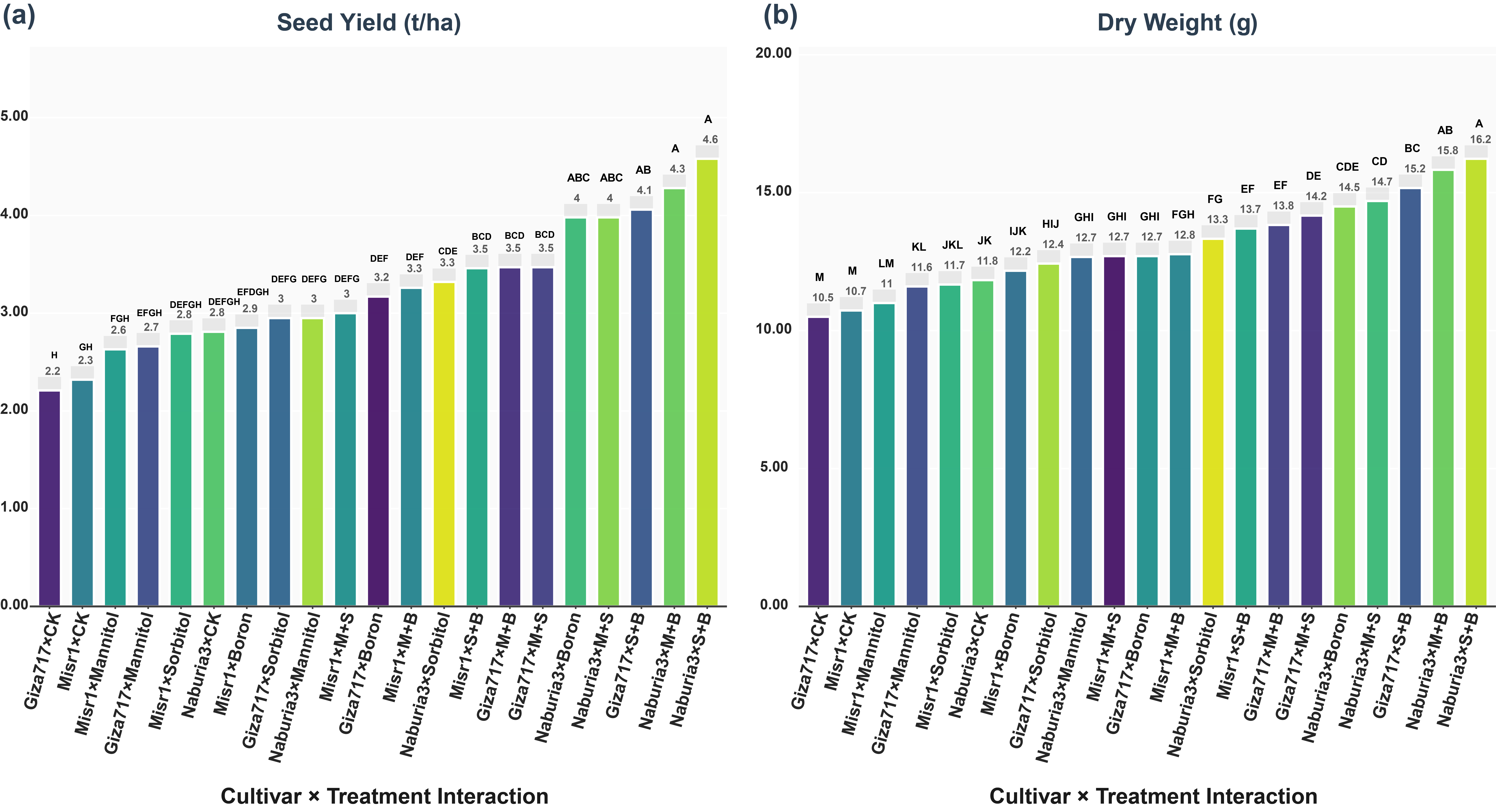


**Figure S3.** Cultivar × treatment interaction effects on yield-related parameters in faba bean during the second growing season. Three cultivars (Giza717, Misr1, Nubaria3) were evaluated under seven foliar treatments: CK (control/water only), M (Mannitol 50 mM), S (Sorbitol 50 mM), B (Boron 0.2%), M+S (Mannitol + Sorbitol), M+B (Mannitol + Boron), and S+B (Sorbitol + Boron). (**a**) Seed yield expressed as tonnes per hectare (t/ha). (**b**) Plant dry weight at maturity expressed in grams (g). Bars represent mean values of the interaction effects (n = 3 biological replicates). Different uppercase letters above bars indicate statistically significant differences among all 21 cultivar × treatment combinations within each parameter based on Tukey's HSD test (p < 0.05). Combinations sharing the same letter are not significantly different from each other.
